# YOLO-UMS: Multi-Scale Feature Fusion Based on YOLO Detector for PCB Surface Defect Detection

**DOI:** 10.3390/s26020689

**Published:** 2026-01-20

**Authors:** Hong Peng, Wenjie Yang, Baocai Yu

**Affiliations:** School of Electronic and Information Engineering, Liaoning Technical University, Huludao 125105, China; penghong@lntu.edu.cn (H.P.);

**Keywords:** multi-scale features fusion, YOLO, PCB detection, network design

## Abstract

Printed circuit boards (PCBs) are critical in the electronics industry. As PCB layouts grow increasingly complex, defect detection processes often encounter challenges such as low image contrast, uneven brightness, minute defect sizes, and irregular shapes, making it difficult to achieve rapid and accurate automated inspection. To address these challenges, this paper proposes a novel object detector, YOLO-UMS, designed to enhance the accuracy and speed of PCB surface defect detection. First, a lightweight plug-and-play Unified Multi-Scale Feature Fusion Pyramid Network (UMSFPN) is proposed to process and fuse multi-scale information across different resolution layers. The UMSFPN uses a Cross-Stage Partial Multi-Scale Module (CSPMS) and an optimized fusion strategy. This approach balances the integration of fine-grained edge information from shallow layers and coarse-grained semantic details from deep layers. Second, the paper introduces a lightweight RG-ELAN module, based on the ELAN network, to enhance feature extraction for small targets in complex scenes. The RG-ELAN module uses low-cost operations to generate redundant feature maps and reduce computational complexity. Finally, the Adaptive Interaction Feature Integration (AIFI) module enriches high-level features by eliminating redundant interactions among shallow-layer features. The channel-priority convolutional attention module (CPCA), deployed in the detection head, strengthens the expressive power of small target features. The experimental results show that the new UMSFPN neck can help improve the AP50 by 3.1% and AP by 2% on the self-collected dataset PCB-M, which is better than the original PAFPN neck. Meanwhile, UMSFPN achieves excellent results across different detectors and datasets, verifying its broad applicability. Without pre-training weights, YOLO-UMS achieves an 84% AP50 on the PCB-M dataset, which is a 6.4% improvement over the baseline YOLO11. Comparing results with existing target detection algorithms shows that the algorithm exhibits good performance in terms of detection accuracy. It provides a feasible solution for efficient and accurate detection of PCB surface defects in the industry.

## 1. Introduction

With the development of science and technology, the degree of sophistication of modern equipment is becoming higher and higher. PCB is gradually moving towards miniaturization and complexity of the direction of development, and the continuous enhancement of process requirements may be accompanied by a series of defects in the PCB-manufacturing process, missing holes, mouse bites, open circuits, short, spur, spurious copper, and other defects. These defects may cause PCB performance degradation, and the quality of subsequent products negatively affects the economic losses. Therefore, it is crucial to detect defects on the PCB surface efficiently. Currently used for PCB surface defect detection, the central inspection methods are manual visual inspection method and electrical characteristics of the identification method of destructive testing, non-destructive testing X-Ray detection, infrared thermography, and ultrasonic detection [1,2]. However, constrained by material and environmental factors, both methods are difficult to adapt to different production environments.

Deep learning-based computer vision technology is advancing rapidly due to its cost-effectiveness, high efficiency, and resilience to variations in material and production environments. Consequently, it has been extensively applied in the detection of surface defects on PCB. Detection algorithms in this domain are generally categorized into two types: one-stage and two-stage detection algorithms. A prominent example of two-stage detection algorithms is the RCNN series [3,4,5,6], which conducts target detection through a two-step process. Initially, several candidate regions are generated, followed by the selection of the most accurate regions for localization and classification predictions. Despite their effectiveness, these two-stage algorithms require extensive computational resources, leading to slower inference speeds and lower frame rates, particularly on devices with limited processing capabilities. In contrast, one-stage detection algorithms—including the YOLO series [7,8,9,10], SSD [11], and FCOS [12]—approach the target detection task as a single machine learning challenge, generating predictions directly from the input image without relying on candidate regions. These methods not only maintain high levels of accuracy but also preserve the benefit of rapid detection, making them particularly well-suited for real-time applications in intricate environments, and they are especially advantageous for detecting defects on PCB surfaces.

Related research work has further improved the generalized detector to enable the model to recognize multiple defects efficiently and accurately simultaneously to achieve a better trade-off between accuracy and speed. Ref. [13] introduced inverted residual blocks and coordinate attention [14] in the YOLOX framework [15], effectively reducing the network parameters while enhancing the recognition of PCB surface defects. Ref. [16] Based on YOLOv7 [17], FasterNet [18] and the Convolutional Block Attention Module (CBAM) [19] are combined to effectively extract spatial features, reduce redundant operations, and enhance the discriminative ability of feature expression to improve detection accuracy. Ref. [20] designed LDD-Net, a lightweight PCB surface defect detection network that focuses on critical defect features through efficient downsampling and attention module.

Although these methods have achieved relatively good results in PCB surface defect detection, there are still some challenges: (1) Reducing the network’s weight may impact its accuracy without necessarily improving the model’s real-time performance. This is because hardware conditions limit the model’s inference speed, and excessive reliance on low computational resources can impede the model’s deployment on GPUs. (2) The increasingly small size of PCB surface defects and the interference of complex backgrounds pose significant challenges to the target detection capability of existing models. Most existing methods make it difficult to fuse multi-scale features, resulting in noticeable intra-class differences and inter-class similarities. (3) Although the introduction of attention modules, such as CBAM and SE, can reduce the unnecessary interference of noise for model detection to a certain extent, complex environments that include light variations in production environments, dust, and other interfering factors may lead to blurring of defect boundaries, which affects the accuracy of defect detection on PCB surfaces.

To achieve lightweight without affecting the detection accuracy and further improve the model’s performance, an RG-ELAN module combining the ideas of ELAN and GhostNet [21] is designed, using a heavily parameterized module as the computational block. The introduction of the Adaptive Interaction Feature Integration (AIFI) [22] module captures the dependencies between features. It removes unnecessary interactions between shallow features, enabling the model to process and fuse important information more effectively, thus further improving the overall detection performance.

To mitigate the uncertainty caused by smaller defect sizes and complex backgrounds, this paper introduces a multi-branching neck architecture for multi-scale feature fusion, namely the Unified Multi-scale Feature Fusion Pyramid Network (UMSFPN). A key component of this network is the Cross Stage Partial Multi-scale Module (CSPMS), specifically designed to enhance feature representation across different scales. This neck framework improves detection accuracy and performance for small target detection by effectively fusing multi-scale features. It is particularly well-suited for application scenarios with complex scenes and small targets’ extensive, dense distribution. On a custom dataset, UMSFPN achieves a 2% improvement in AP and a 3.1% improvement in AP50, outperforming the original neck structure. Additionally, it is plug-and-play with other deep learning models and demonstrates exceptional generalization across different datasets.

The channel-priority convolutional attention module (CPCA) [23], which can dynamically allocate attention weights in channel and spatial dimensions, is added to the detection head of the model to cope with the problem of the model’s insufficient detection ability for small targets in complex environments. As a result, the proposed YOLO-UMS model achieves a superior trade-off between speed and accuracy compared to existing detectors.

The main contributions of this paper are summarized as follows:

(1) Aiming at the problems of complex backgrounds, small-size defects and diversified features in PCB surface defect detection, the RG-ELAN feature extraction method is proposed. This method effectively reduces the uncertainty in the detection process and improves the detection accuracy of the small target information while realizing lightweight. The AP50 is improved by 4.1% on the PCB-M dataset while reducing the computation amount and realizing lightweight.

(2) The AIFI module is introduced to replace the traditional SPPF module, which reduces shallow feature interactions, optimizes deep semantic information fusion, and improves the detection accuracy of small targets.

(3) The UMSFPN is proposed, and by introducing the weighted bi-directional feature pyramid network (BiFPN),the Efficient up-convolution block (EUCB), and designing a new type of feature extraction module CSPMS, the effectiveness and efficiency of feature fusion and extraction are significantly improved. Extraction. At the same time, its plug-and-play modular design and lightweight features make it widely applicable and high-performance in practical applications.

(4) The head network is added with the CPCA to reduce the influence of uncertainty factors on the model and further enhance the granularity of small target features.

## 2. Related Works

### 2.1. PCB Surface Defect Detection

PCB is a core component of electronic devices, and its quality directly affects electronic products’ performance and service life. With the rapid development of deep learning technology, applying related techniques in PCB surface defect detection significantly improves the accuracy and robustness of the system. Unlike two-stage inspection methods, single-stage inspection methods do not require deep extraction of candidate regions, thus increasing the inspection speed, especially in real-time inspection, demonstrating its unique advantages.

The YOLO series of detection frameworks is widely used in industrial inspection due to its good balance between accuracy and speed. Ref. [7] introduced Cross Stage Partial Network (CSPNet) [24] and Mish activation function as the backbone network in YOLO, which reduces the amount of computation, and through spatial pyramid pooling (SPP) [25] with a multiscale prediction path Aggregation Network (PANet) extends the sensory field, which ensures the inference speed and improves the performance at the same time. YOLOv5, on the other hand, employs the SPPF layer [26], which accelerates the computation by aggregating the features of different scales into a fixed-size feature map. YOLOX employs an advanced label assignment strategy and powerful data enhancement techniques to achieve an optimal balance. YOLO6 [27] network design combines an efficient backbone of RepVGG blocks [28], a PAN topology neck, and an efficient decoupling header with a hybrid channel strategy. The architecture of YOLOv7 draws on the reparameterized convolution (RepConv) technique and proposes the E-ELAN module for network models with infinitely stacked computational blocks. YOLOv8 is optimized based on YOLOv6 and YOLOv7, focusing more on engineering practices. The latest YOLO11 model builds on YOLOv8 by introducing the C3k2 (cross-stage part of kernel size 2) block, SPPF, and C2PSA (convolutional block with parallel spatial attention) components, which improve the feature extraction capability of the model in several ways.

In addition to the direct application of the YOLO model models for PCB surface defect detection, several improvement schemes are aimed at coping with more complex situations in PCB inspection. Ref. [29] replaced the traditional convolutional and ELAN modules in YOLOv7-tiny with deformable convolution. They combined them with the SE (Squeeze-and-Excitation) attention to enhance the extraction capability of small defect features in complex environments. Ref. [30] proposed a CDI-YOLO algorithm based on the improved YOLOv7-tiny, which enhances the feature extraction capability by introducing the CA, employs the DSConv to reduce the computational cost and accelerates the detection speed, and finally accelerates the detection speed by the Inner-CIoU to accelerate the bounding box regression, which improves the detection effect. However, the robustness of the model still needs to be further optimized. Ref. [31] proposed SWS-YOLOv8n as the basis of the YOLOv8 model [32], which improves the fusion and extraction capability of feature information by introducing the SA module, and changes the loss function to a dynamic non-monotonic loss function, which enhances the model’s key pixels, in addition to adopting a non-step convolutional layer to solve the problems of loss of fine-grained defect location information and low learning efficiency, thus improving the detection accuracy. Nevertheless, these approaches do not succeed in obtaining a beneficial balance between speed and precision. Ref. [33] proposed LDM-PCB to address long-tail distribution and multi-scale challenges, utilizing an adaptive tail attention module and a dynamic feature fusion architecture to enhance small defect capture. This study introduces the RG-ELAN backbone feature extraction module, which is built upon the YOLO11 [34] base model, aimed at improving the ability to extract features from small targets. Meanwhile, a new neck structure, UMSFPN, is proposed to utilize the information of different feature levels fully, and the AIFI module is introduced to enhance the feature interaction capability. In addition, the CPCA is incorporated to enhance the model’s ability to capture detailed information and thus improve detection performance.

### 2.2. Multi-Scale Feature Fusion

Multi-scale feature fusion is a pivotal mechanism in object detection, designed to bridge the semantic gap between high-level features and low-level spatial details. In PCB defect detection, where defects range from microscopic “pinholes” to relatively large “spurs,” a robust fusion strategy is essential for achieving scale-invariant detection.

The inception of Feature Pyramid Networks (FPNs) [35] marked a milestone by introducing a top–down pathway to propagate rich semantic information to shallow layers. To further address the loss of localization cues, PANet [26] introduced a complementary bottom–up augmentation path, ensuring that low-level structural features reach the prediction heads. Subsequent models, such as YOLOv4 and YOLOv5, adopted the CSP-PANet structure to balance computational efficiency with feature richness. However, these structures typically rely on simple concatenation or addition, which implicitly assumes that all feature levels contribute equally to the final prediction—a premise that is often invalid in complex PCB backgrounds.

To overcome the limitations of static fusion, recent research has pivoted towards adaptive mechanisms. BiFPN [36] introduced learnable weights for each scale, allowing the network to selectively focus on the most informative resolutions. Similarly, ASFF [37] utilized a spatial-aware weighting strategy to suppress contradictions across scales. In the context of the YOLO lineage, YOLOv6-v3 [38] and DAMO-YOLO [39] explored bidirectional cascades (BICs) and reparameterized generalized FPNs (RepGFPN) to optimize the information flow. Despite their effectiveness, these methods often introduce significant latency on edge devices due to dense skip connections or complex weighting operations.

Modern fusion strategies have increasingly integrated global context to assist local feature extraction. For instance, Gold-YOLO [40] leverages a Gather-and-Distribute mechanism to facilitate global–local interactions. Furthermore, methods like AFPN [41] propose asymptotic fusion to reduce the aliasing effect during cross-layer integration. However, these complex architectures are often overkill for real-time PCB inspection where the trade-off between speed and precision is extremely tight.

While the aforementioned methods have advanced the field, they often struggle with two specific challenges: (1) the excessive computational overhead of dense fusion paths [42], and (2) the blurriness introduced by standard upsampling operations when recovering tiny defect features. To address these, this paper proposes the UMSFPN (Universal Multi-Scale Feature Pyramid Network). Our approach integrates the weighted fusion logic of BiFPN with an Efficient Upsampling Convolutional Block (EUCB) [43], which reconstructs high-resolution features with minimal noise. Furthermore, by incorporating the CSPMS (Cross Stage Partial Multi-scale) module, we facilitate a more granular multi-branch interaction, allowing the model to capture subtle defect patterns that are typically lost in conventional FPN structures.

## 3. Methods

### 3.1. Overall Network Structure

**Figure 1:**
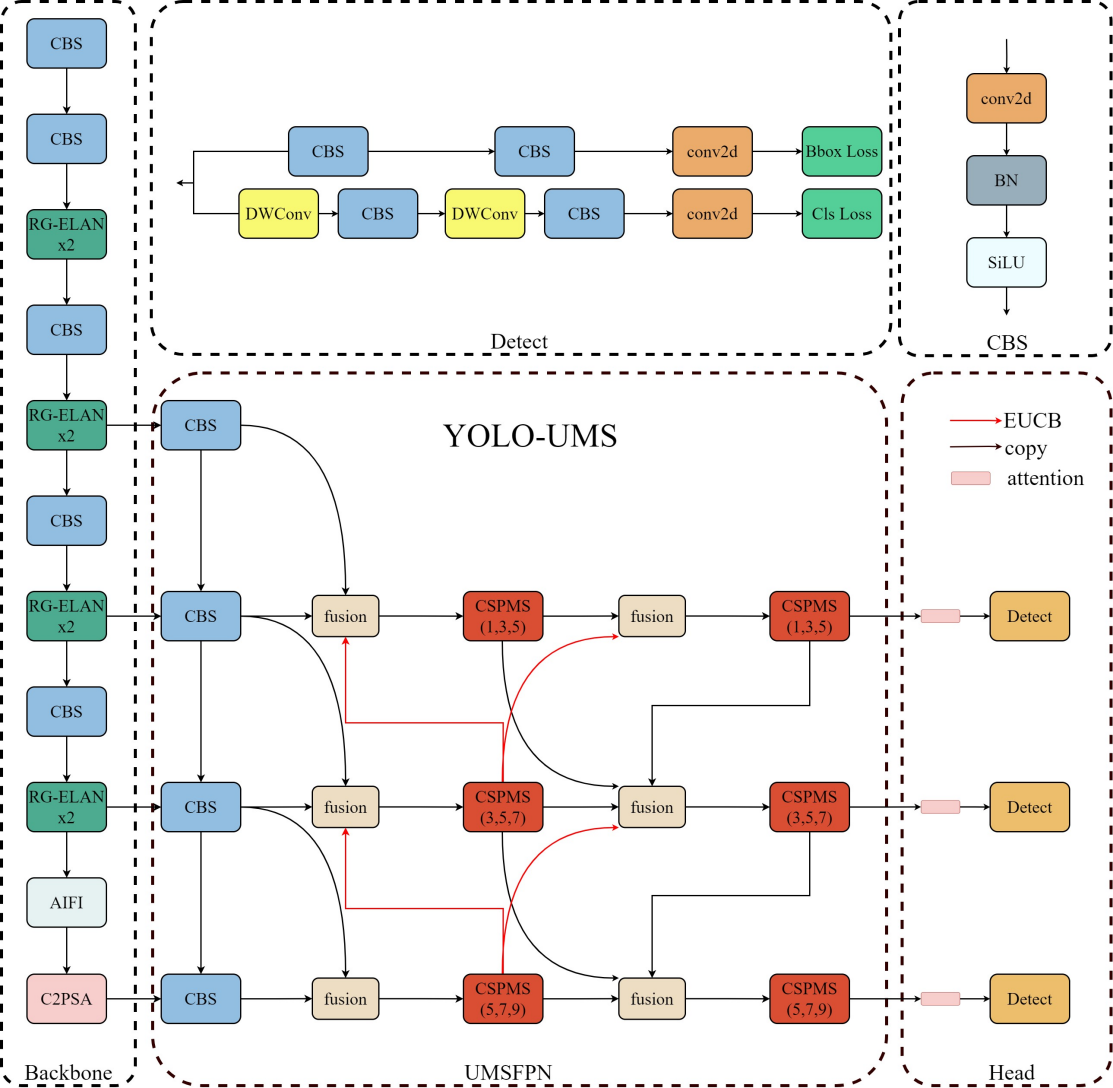
Overall structure of YOLO-UMS.

In this paper, YOLO11 [34] is used as the base model, the state-of-the-art target detector suitable for this paper’s research on PCB surface defect detection with considerable accuracy and speed. On this basis, designing the RG-ELAN feature extraction module and the new UMSFPN neck, as well as introducing the AIFI module and the CPCA, the YOLO-UMS model is proposed, and its overall structure is shown in Figure 1.

YOLO-UMS consists of three main components: the backbone, neck, and head. In the backbone, features are downsampled and extracted using 3 × 3 convolutions to preserve pixel information. The network is structured into four stages, each employing a 3 × 3 convolution for downsampling and channel expansion. Feature extraction is enhanced through a combination of 3 × 3 convolutional downsampling and RG-ELAN, balancing detail retention and model efficiency, with two RG-ELANs per stage. Following the fourth stage, AIFI is integrated to expand the multi-scale receptive field, while C2PSA refines features. The UMSFPN neck facilitates multi-scale feature fusion across multiple branches, where CSPMS strengthens multi-scale sensing and enhances feature extraction. Additionally, BiFPN’s weighted fusion method effectively processes features of varying scales. For detection, the head predicts small targets on high-resolution feature maps and large targets on low-resolution ones. Building on the YOLO11 detector head, the improved design incorporates the CPCA, leveraging semantic information from the backbone and neck for more precise predictions.

### 3.2. RG-ELAN

PCB surface defect detection presents challenges due to complex backgrounds, small defect sizes, and diverse feature variations. Traditional network feature extraction methods struggle to capture these tiny defects effectively, leading to poor performance. To solve this problem, the RG-ELAN module is designed to enhance the feature extraction capability of small targets and reduce the computation and storage consumption, considering the lightweight requirement.

**Figure 2:**
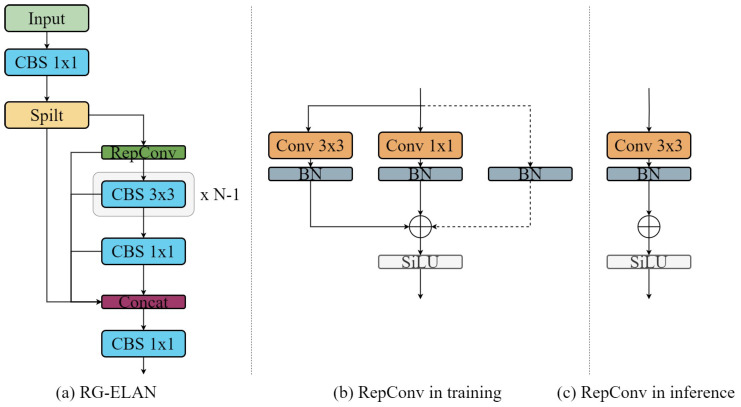
RG-ELAN.

This module is built on the ELAN architecture. By integrating RepConv [44] and GhostNet concepts, it efficiently extracts lightweight features. The RepConv layer, which is parameterizable, enhances the expressive ability of the network through multi-branching structures in the training phase; in the inference phase, these multi-branching structures are merged into a single standard convolutional layer, which reduces the computational complexity and the model parameters and improves the inference speed. ELAN is a high-efficiency layer-aggregation network architecture aimed at optimizing the fusion of the features of the different layers of neural networks and the transmission mechanism to improve the model’s overall performance. The transfer mechanism improves the overall performance and efficiency of the model. Specifically, RG-ELAN introduces the RepConv layer based on ELAN and combines the design concepts of GhostNet to generate more feature maps by cheap 1 × 1 convolution operation. RG-ELAN is used to improve the feature extraction in the backbone part, which reduces the amount of computation and realizes the lightness, as shown in Figure 2.

In the RG-ELAN module, 1 × 1 convolution is first used to increase the number of input channels to twice the number of channels in the hidden layer, which facilitates the extraction of richer abstract features and prepares for subsequent feature segmentation and processing. Subsequently, a Split operation is performed to divide the input feature map into two branches regarding channel dimension, each with half the number of channels of the original feature map. One branch passes the feature map directly to the output, and the other is processed by the RepConv layer and multiple 3 × 3 convolutional layers for production. Referring to the design concept of GhostNet, the intermediate feature maps in mainstream CNNs tend to have more redundancy. To reduce this redundancy, after N-1 3 × 3 convolutional layers, the branch uses cheap 1 × 1 convolution to generate a portion of new feature maps. With this segmentation, the model can process the feature maps of each branch independently. Subsequently, the features of each branch are merged using a cross-stage hierarchy to ensure that the gradient information in different network paths can be propagated with significant correlation differences, thus enhancing the overall feature extraction capability.

Figure 2b,c show the network structure of RepConv in the training and inference phases. Jump connections model the information flow to enhance the feature extraction capability. The reparameterization process is described as follows: in the training phase, the inputs are batch normalized after being computed through three branches of constant connections, 3 × 3 convolutional layers and 1 × 1 convolutional layers, followed by element-by-element summation and passing through the activation function layer to obtain the results.

(1) $$\begin{array}{cc} M_{\mathrm{out}}\; & =BN\left(M_{\mathrm{in}},\lambda_{\mathrm{id}},\eta_{\mathrm{id}},\gamma_{\mathrm{id}},\beta_{\mathrm{id}}\right)\\ \; & +BN\left(M_{\mathrm{in}}*W_{k1},\lambda_{k1},\eta_{k1},\gamma_{k1},\beta_{k1}\right)\\ \; & +BN\left(M_{\mathrm{in}}*W_{k3},\lambda_{k3},\eta_{k3},\gamma_{k3},\beta_{k3}\right) \end{array}$$

$M_{in}$ and $M_{out}$ are the input–output feature maps, and $W_{k1}$ and $W_{k3}$ denote the weights of the 1 × 1 and 3 × 3 convolutions, respectively. $\lambda_{k3}$, $\eta_{k3}$, $\gamma_{k3}$, and $\beta_{k3}$ are the BN’s mean, standard deviation, learning scale factor, and bias accumulation after 3 × 3 convolution. Similarly, $\lambda_{k1}$, $\eta_{k1}$, $\gamma_{k1}$, and $\beta_{k1}$ denote 1 × 1 convolution, while $\lambda_{id}$, $\eta_{id}$, $\gamma_{id}$, and $\beta_{id}$ are used for constant branching. Constant branching is applicable only when the input and output channel dimensions are identical, and the convolution stride is 1. In this case, it functions as a 1 × 1 branch with a fixed weight of 1. A 1 × 1 convolution, in turn, can be viewed as a zero-padded 3 × 3 convolution. Hence, the 3 × 3 branch is used to illustrate the reparameterization process:

(2) $$\begin{array}{c} BN(M_{n}*W_{k3},\lambda_{k3},\eta_{k3},\gamma_{k3},\beta_{k3})\\ =\left(M_{n}*W_{k3}-\lambda_{k3}\right)\frac{\gamma_{k3}}{\eta_{k3}}+\beta_{k3}\\ =M_{n}*\left(\frac{W_{k3}\gamma_{k3}}{\eta_{k3}}\right)-\frac{\lambda_{k3}\gamma_{k3}}{\eta_{k3}}+\beta_{k3} \end{array}$$

Thus, the weights and biases of the 3 × 3 branch can be converted into $W_{k3}^{*}=\frac{W_{k3}\gamma_{k3}}{\eta_{k3}}$ and $b_{k3}^{*}=-\frac{\lambda_{k3}\gamma_{k3}}{\eta_{k3}}+\beta_{k3}$. The other two branches can be converted into A and B similarly using the 3 × 3 branch. A similar derivation can be obtained for the other two branches. The final output of RepConv in the inference process is expressed as follows:

(3) $$\begin{array}{cc} \;\;\;\;\;\;\;\;M\; & =\mathrm{SiLU}\left(M_{\mathrm{out}}\right)=\mathrm{SiLU}\left(M_{\mathrm{in}}*W^{*}+b^{*}\right) \end{array}$$

(4) $$\begin{array}{cc} W^{*}\; & =W_{k3}^{*}+W_{k1}^{*}+W_{\mathrm{id}}^{*} \end{array}$$

(5) $$\begin{array}{cc} b^{*}\; & =b_{k3}^{*}+b_{k1}^{*}+b_{\mathrm{id}}^{*}\; \end{array}$$

where *M* represents the output of RepConv with the SiLU activation function, and $W_{k1}^{*}$ and $W_{id}^{*}$ are the weights of 1 × 1 convolution and constant branch, respectively; $b_{k1}^{*}$ and $b_{id}^{*}$ are the deviations of 1 × 1 convolution and constant branch, respectively. The multi-branch architecture allows the model to be viewed as a collection of multiple shallower models, effectively improving the network representation and preventing gradient vanishing.

In the inference phase, the method reduces the three-branch structure in the training phase to a combination of a 3 × 3 convolutional layer and an activation function layer through a constant transform, which maintains the feature extraction capability and reduces the computational complexity. The single-branch structure improves parallelism and inference speed while increasing the nonlinear and representational capabilities of the network and enhancing the ability to model complex data. The process merges all branches into a single convolutional kernel, making the inference phase more computationally efficient while maintaining the performance advantage of the training phase.

Ultimately, the reparameterization process enables the model to achieve a single-branch structure in the inference phase. This significantly improves parallelism and inference speed while retaining the multi-branch design’s representational capabilities and nonlinear advantages in the training phase.

### 3.3. AIFI

AIFI can reduce unnecessary interactions between networks, thus effectively reducing cross-scale attention computation overhead. Introducing the AIFI module into YOLO11 enhances interaction with intra-scale features. It works with the UMSFPN neck to further improve the effect of multi-scale feature fusion, thus obtaining richer global features. Compared with SPPF, AIFI not only enhances the feature interaction capability and optimizes the computational overhead but also adopts dynamic adjustment of the fusion method to improve the generalization capability and adaptability of the model, better work with complex network structures (e.g., UMSFPN), and improve the overall performance.

**Figure 3:**
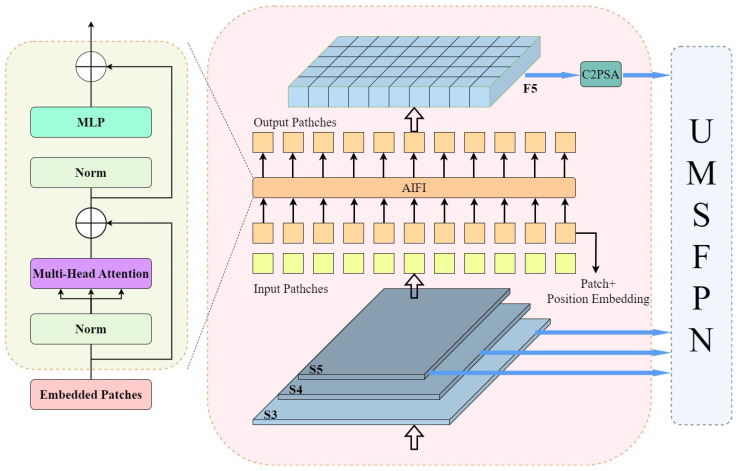
Internal structure diagram of AIFI.

The feature fusion process of the AIFI module is shown in Figure 3, where the two-dimensional S5 feature maps are first converted into high-dimensional vectors by linear transformation or convolution operation to accommodate the subsequent self-attention mechanism. Subsequently, these high-dimensional vectors are input into the Multi-Head Self-Attention module, where multiple attention heads capture the complex dependencies between features in parallel to fully understand the feature map’s information. The output of Multi-Head Self-Attention is summed up with the original input through residual linkage. It undergoes Layer Normalization, effectively alleviating the gradient vanishing problem and stabilizing the training process. Then, the feature information is passed to the Feed-Forward Neural Network for nonlinear transformation and feature extraction, further enhancing the feature expression ability. After another layer of normalization, attention scores containing important information are generated, reflecting each feature’s importance in the global context. Finally, the processed features are converted to a 2D form, denoted as F5, for subsequent multi-scale feature fusion. The AIFI module only processes the S5 feature map because it has more advanced semantic information than the shallower S3 and S4 feature maps, which allows for more effective differentiation of different objects while at the same time reducing the amount of computation and has a minimal impact on the detection performance.

In addition, combining the AIFI module with UMSFPN and RG-ELAN further optimizes the fusion process of the multi-scale features. It enhances the ability to capture the dependencies between the features, thus significantly improving the overall detection performance. Through this kind of teamwork, AIFI not only outperforms the traditional SPPF module in terms of feature interaction and computational efficiency but also improves the generalization ability and adaptability of the model by dynamically adjusting the fusion strategy, which makes the model perform even better in complex environments.

### 3.4. UMSFPN

In the target detection task, multi-scale feature fusion can effectively combine different levels of feature information to enhance the detection effect. Classical feature fusion architectures such as Feature Pyramid Networks (FPN) and Path Aggregation Networks (PAN) have been widely used in YOLO detectors. However, when performing multi-scale (especially small target) detection in complex scenes, traditional FPN and PAN have certain limitations regarding efficient computation and high-quality feature fusion.

This paper proposes a plug-and-play neck, UMSFPN, to address these issues. The main features include the following: (1) Multi-scale weighted feature fusion: the weighting mechanism of BiFPN is introduced to dynamically adjust the weights of features at different levels, making multi-scale feature fusion more adaptive. (2) Efficient up-sampling mechanism: A lightweight EUCB module is designed to optimize the spatial resolution recovery of shallow features. (3) Feature extraction module optimization: The newly designed CSPMS module improves the efficiency and diversity of feature extraction by multi-scale convolution and global heterogeneity mechanisms.

Uniform channel number design: In designing the new neck, the same number of channels is used in the convolutional layers from the output of the backbone network to each branch of the neck, and the channel number of the whole neck network is uniformly 256.

#### 3.4.1. Using BiFPN’s Weighted Feature Fusion Method

A single multi-scale feature representation falls short of capturing the complexity of PCB surface defects, which are diverse in morphology and size and are affected by noise, illumination variations, and background interference. Although traditional shallow and deep feature fusion methods can improve detection accuracy, it is difficult for them to simultaneously meet the demands of coping with complex defects in terms of efficiency and accuracy. The UMSFPN designed in this study uses a BiFPN to assist in fusing shallow and deep feature information.

The introduction of the BiFPN structure significantly enhances the network sensing capability, connecting high-level semantic and low-level spatial information through cross-layer feature fusion. It excels in small-target detection (e.g., tiny defects). The bidirectional information flow design of BiFPN ensures that the low-level fine-grained features (e.g., small-target edges and details) are effectively retained in the multilayer processing. Meanwhile, the deep-layer high-semantic information captures global features of large targets (e.g., more significant defects or anomalous regions) to further optimize the tiny defect detection capability.

The weighted feature fusion mechanism dynamically adjusts the importance of input features to maximize their utility. Specifically, BiFPN assigns learnable weights to each feature and normalizes them using a method similar to SoftMax, which scales the weights to a range of [0, 1] and ensures they sum to 1. This normalization plays a key role in constraining and stabilizing the weights during training. The formula for fast normalized fusion is shown below:

(6) $$\begin{array}{cc} O & =\sum_{i=0}\frac{\omega_{i}}{\varepsilon +\sum_{j=0}\omega_{j}}\cdot I_{i} \end{array}$$

where $\omega_{i}$ is the learnable weight, the activation function ReLU ensures that each $\omega_{i}\geq 0$ can be obtained from the network training, and the sum of $\omega_{i}$ weights is first calculated. Then, a small constant ε=0.0001 is added for numerical stabilization. Then, the weights are normalized. Finally, the input features $I_{i}$ are weighted using the normalized weights to obtain the output features O.

**Figure 4:**
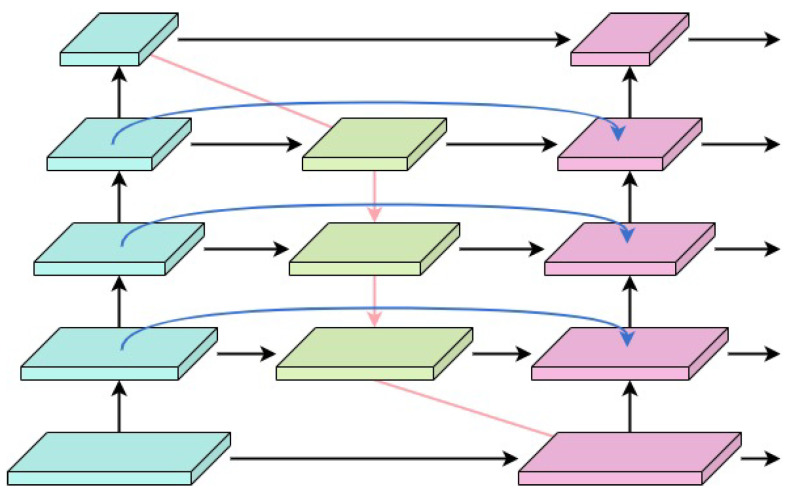
Structure of BiFPN.

As shown in Figure 4, the structure diagram of BiFPN demonstrates its bidirectional feature fusion path. Through this design, UMSFPN not only improves the network’s multi-scale learning capability but also ensures high accuracy under complex interference and background changes.

#### 3.4.2. Feature Fusion Module CSPMS

A new feature fusion module, CSPMS, has been designed to fulfill the UMSFPN feature extraction and fusion capability. CSPMS integrates a CSP structure and replaces the traditional C2f residual block with an MSCB block.

**Figure 5:**
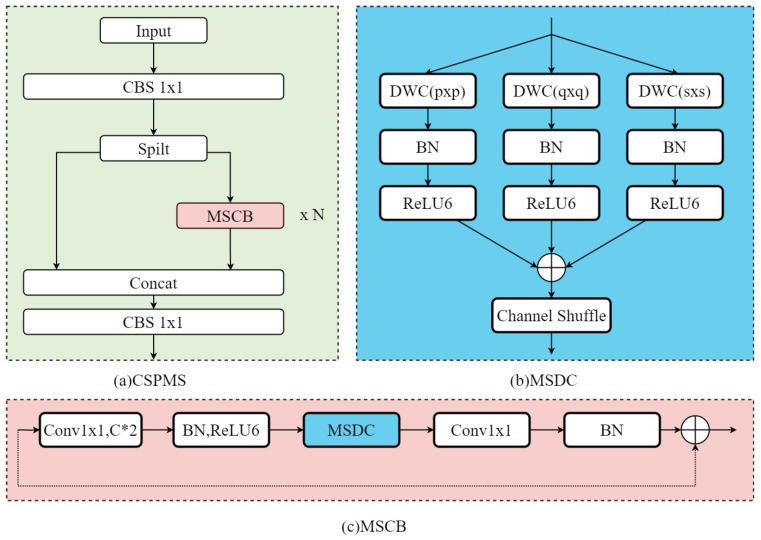
Structure of CSPMS.

The CSP structure splits the input feature maps into two parts, one of which is passed directly to the subsequent layers. In contrast, the other part is merged with the original feature maps after multiple convolution operations. This design effectively reduces redundant computations, maintains feature diversity, and significantly improves computational efficiency. Specifically, the CSP structure ensures the retention of key information by directly transferring some of the features while enhancing the feature expression capability through convolutional processing. Figure 5a illustrates the detailed structure of the CSPMS, showing how the feature map is segmented and processed by multiple MSCB blocks before merging. This design reduces redundant computations, preserves feature diversity, and improves computational performance.

Since networks with larger receptive fields are suitable for detecting large objects, while small objects benefit from smaller receptive fields, the UMSFPN extends the Global Heterogeneous Kernel Selection (GHKS) mechanism by integrating heterogeneous convolutional kernels. In CSPMS, convolution kernels of different sizes, 1 × 1, 3 × 3, 5 × 5, 7 × 7, and 9 × 9, are used to adapt to different resolutions and to acquire multi-scale perceptual information progressively.

Multi-scale deep convolution (MSDC) is the core component of CSPMS, and its structure is shown in Figure 5b. MSDC collects information from multiple perceptual fields using convolutional kernels of different sizes in parallel to capture rich contextual details. Each convolutional kernel extracts features at various spatial scales, enabling the network to extract fine-grained features from multiple receptive fields and enhance the perception of multi-scale targets. The output of the MSDC is adaptively fused to reduce redundancy and improve the interactions between features. Subsequently, the feature channels are reorganized by Channel Shuffle to optimize the information flow and make the information fusion at different scales more efficient.

The following equation can describe the computational process of MSDC:

(7) $$\begin{array}{c} MSDC\left(x\right)=\sum DWCB_{KS}\left(x\right) \end{array}$$

KS denotes a collection of multi-scale convolutional kernels, $DWCB_{KS}$ denotes deep convolutional blocks with different kernel sizes, and X is the input feature map. Each convolutional kernel processes the feature maps in parallel, capturing contextual information at various scales.

Figure 5c illustrates the structure of the multi-scale efficient convolution module, a key component of CSPMS. This module combines MSDC and pointwise convolution to improve the network’s feature extraction capability by efficiently processing multi-scale features.

The process first extends the number of channels of the input feature map using a pointwise convolution layer (extension factor = 2) to provide richer feature information for subsequent convolution operations, followed by applying batch normalization (BN) and the ReLU6 activation layer. Then, multi-scale deep convolution is performed by the MSDC module to capture the contextual information of different receptive fields to enhance feature diversity and improve the adaptability to targets of different sizes. After multi-scale convolution, the feature map undergoes a channel-shuffling operation to reorganize the feature channels and enhance feature interaction. Next, redundant features are reduced by channel compression to ensure that the computational overhead is controlled while maintaining information richness. Finally, batch normalization (BN) is applied to stabilize the training and enhance the model’s generalization ability. The computational formula for the whole process can be expressed as follows:

(8) $$MSCB\left(x\right)=BN\left(PWC_{2}\left(CS\left(MSDC\left(R6\left(BN\left(PWC_{1}\left(x\right)\right)\right)\right)\right)\right)\right)$$

This multi-scale efficient convolution module (MSCB) significantly improves the model’s robustness and accuracy in handling objects of different sizes by optimizing the feature flow and efficiently extracting multi-scale features. It mainly demonstrates excellent performance in small target detection in complex backgrounds.

By combining MSDC and pointwise convolution (PWC), the CSPMS module can efficiently process feature information at different scales and optimize feature interaction and information flow through channel shuffling and fast normalized fusion strategies. This design significantly improves the model’s robustness and accuracy in multi-scale target detection tasks, especially in small target detection in complex backgrounds. In addition, CSPMS improves overall computational efficiency by reducing redundant computations and preserving feature diversity, which enables UMSFPN to have better detection performance and real-time performance in real applications.

#### 3.4.3. EUCB

**Figure 6:**
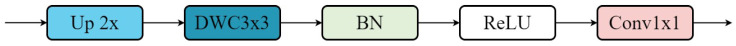
EUCB.

In this study, an EUCB is employed to gradually upsample the feature maps at the current stage to ensure that their size and resolution can match the feature maps of the next jump connection. The up-sampling operation plays a crucial role in feature fusion in networks, especially when dealing with multi-scale features, to effectively recover fine-grained spatial information and ensure that the feature maps do not lose important visual information in the delivery process. EUCB optimizes the up-sampling process of feature maps through a multi-step design to achieve efficient computational performance and accurate feature fusion results, as shown in Figure 6.

To match the size of the feature maps in the subsequent jump connection, EUCB first uses an up-sampling operation (Up) with a scale factor of 2 to double the size of the feature maps in the current stage. The up-sampled feature maps are then subjected to a 3 × 3 Depthwise Convolution (DWConv), which preserves computational efficiency without appreciably raising the computational overhead and allows for the extraction of more local information. Batch Normalization (BN) and ReLU activation functions are used after the convolution operation to further improve the expressiveness of the feature maps and add nonlinear features, which enhances feature learning.

To ensure the feature maps are consistent in terms of the number of channels, the final step of the EUCB utilizes a 1 × 1 convolution (Pointwise Convolution) to reduce the channel count, aligning the final output feature maps with the number of channels in the feature maps of the network’s next stage. This adjustment guarantees that the dimensions of the feature maps at each stage can be seamlessly connected, allowing for effective feature transfer to the subsequent network layers.

The following equation can describe the operation of the formulaic representation EUCB:

(9) $$EUCB\left(x\right)=C_{1x1}\left(\mathrm{ReLU}\left(\mathrm{BN}\left(\mathrm{DWC}\left(\mathrm{Up}(x)\right)\right)\right)\right)$$

X is the input feature map, Up denotes the upsampling operation, DWConv is the depth convolution, BN is the batch normalization operation, and $C_{1x1}$ is the 1 × 1 convolution operation.

### 3.5. CPCA

The CPCA module is added to the YOLOv11 header network to enhance further the model’s ability to detect small-target defects. CPCA is a channel-prioritized convolutional attention module that forms deep spatial attention using multi-scale depth-separable convolutional modules. It dynamically allocates weights in both channel and spatial dimensions, enhancing the representation of small-target-related features and improving detection accuracy. Introducing this prior mechanism ensures more reasonable attention allocation, allowing the network to select feature channels relevant to small targets better, thus improving detection performance. Additionally, applying CPCA across different levels of feature maps further refines the small-target features and boosts detection accuracy.

**Figure 7:**
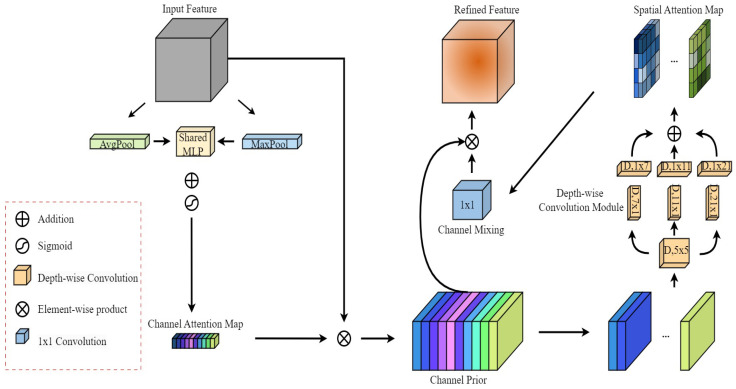
The CPCA structure.

The structure of the channel-prioritized convolutional attention module is shown in Figure 7, which is similar to CBAM and adopts the form of the channel first and space later. Given an intermediate feature map $F\in R^{CxHxW}$, the channel attention module first deduces a one-dimensional channel attention map $M_{C}\in R^{Cx1x1}$ and multiplies element-by-element $M_{C}$ with the input feature F. Then, it broadcasts the channel attention values along the spatial dimension to obtain the fine features with channel attention $F_{C}\in R^{CxHxW}$. The spatial attention module processes $F_{C}$ to generate a three-dimensional spatial attention map $M_{S}\in R^{CxHxW}$. The final output feature, $\hat{F}\in R^{C\times H\times W}$, is obtained by multiplying $M_{S}$ and J$F_{C}$ element by element. The final output feature, F, is obtained by multiplying H and J element by element. The total attention process can be summarized follows:

(10) $$\begin{array}{cc} F_{c}\; & =\mathrm{CA}(F)⊗F \end{array}$$

(11) $$\begin{array}{cc} \hat{F}\; & =\mathrm{SA}\left(F_{c}\right)⊗F_{c} \end{array}$$

where ⊗ denotes element-by-element multiplication.

Average pooling and global maximum pooling operations are used to aggregate the spatial information in the feature map. This aggregation process produces two separate spatial context descriptors. These descriptors are then fed into a shared multilayer perceptron (MLP) and a sigmoid activation function to compute the weights for each channel. The channel attention maps are obtained by combining the outputs of the shared MLP in an element-by-element summation. To reduce parameter overhead, the shared MLP consists of a single hidden layer where the size of the hidden activation is set to $R^{C/rx1x1}$, and r denotes the reduction ratio. The computation of channel attention can be summarized as follows:

(12) $$\mathrm{CA}\left(F\right)=\sigma \left(\mathrm{MLP}\left(\mathrm{AvgPool}(F)\right)+\mathrm{MLP}\left(\mathrm{MaxPool}(F)\right)\right)$$

where σ denotes the sigmoid function.

To avoid enforcing consistency in the spatial attention map for each channel, it is considered more realistic to dynamically assign attention weights in both channel and spatial dimensions. Figure 7 illustrates the use of deep convolution to capture spatial relationships between features, ensuring that inter-channel relationships are preserved while reducing computational complexity.

Spatial attention captures spatial features at different scales through depth-separable convolutional blocks of various sizes. Then, based on spatial convolution, the features are further refined through 1 × 1 convolution to achieve channel mixing of features. Finally, the weights of channel and spatial attention are applied to the original features to realize the weighting and reconstruction of the features. The calculation of spatial attention can be described as follows:

(13) $$\mathrm{SA}\left(F\right)={\mathrm{Conv}}_{\mathrm{lvl}}\left(\sum_{i=0}^{3}{\mathrm{Branch}}_{i}\left(\mathrm{DwConv}(F)\right)\right)$$

DwConv denotes the depth convolution, $Branch_{i},i\in \left\{0,1,2,3\right\}$ denotes the ith branch, and $Branch_{0}$ is the identity connection. A strip convolution in both depth directions approximates a standard per-channel convolution with a large kernel. The size of the convolution kernel is different for each channel to capture multi-scale information.

## 4. Experiments and Results

### 4.1. Implementation Details

#### 4.1.1. Dataset

This paper uses a self-collected dataset for model comparison and ablation experiments to fully validate the proposed method’s effectiveness and generalization ability. Two popular public datasets are also chosen to test the model’s generalization performance.

The first dataset, PCB-M, is expanded based on the publicly available PCB surface defect dataset released by the Human–Computer Interaction Open Laboratory of Peking University (PKU-Market-PCB). The original PKU-Market-PCB dataset contains 1386 images covering six types of defects: spur, mouse bite, short, spurious copper, missing hole, and open circuit. To enhance the diversity of the PCB-M dataset, image enhancement techniques, such as horizontal and vertical flipping, hue and saturation adjustments, and exposure adjustments, were applied to generate 3357 enhanced images.

The second dataset is the publicly available PCB Defect-Augmented (hereafter called PCB-B). Based on PKU-Market-PCB, this dataset is designed to comprehensively assess domain adaptation, generalization ability, and robustness. Various image enhancement techniques are applied to the six defects present in the dataset, expanding the original 1386 images to 10,886. This dataset is widely used in defect detection research and is a publicly available standard dataset.

The third dataset is DEEP-PCB (hereafter called PCB-D), in which the images are all obtained from a linear scanning CCD with a resolution of approximately 48 pixels per millimeter. The defect-free template images were manually cleaned and inspected from the sampled images to construct the defective templates. The original size of the template and test images was approximately 16k × 16k pixels, which were cropped to produce several 640 × 640-sized sub-images. The dataset contains the same 6 categories of defects as the PCB-M dataset, totaling 1500 images.

All the datasets are divided into training sets at 80%, 10% as a validation set, and the rest used for testing.

#### 4.1.2. Training Setting

The training strategy is similar to that of YOLOv5, and the same hyperparameters are used for all the deep learning methods involved in the experiments. Although hyperparameter optimization can be very helpful in improving the model, this paper focuses on proposing a new PCB surface defect detection model rather than optimizing the parameters. Table 1 contains a list of the hyperparameter settings used in this work.

The operating system used in this experiment is Windows 11. The CPU is an Intel Core i7-14650HX (Santa Clara, CA, USA), the GPU is an NVIDIA GeForce RTX 4090 (Santa Clara, CA, USA), and the graphics card has 24 GB of memory. The deep learning framework used is Pytorch 2.5.0, which utilizes CUDA 12.4. The programming language is Python 3.10.15.

The initial learning rate is set to 0.01, the model is trained on the above three datasets for 300 cycles, the batch size is 64, and the image size is resized to 640 × 640.

#### 4.1.3. Evaluation Metrics

To evaluate the proposed method, precision, recall, average precision (AP), floating-point operations (FLOPs), and inference speed are used as performance metrics. Precision measures the proportion of correctly classified positive samples among all detected positives, while recall represents the proportion of detected positive samples among all actual positives. Their definitions are as follows:

(14) $$\mathrm{Precision}=\frac{\mathrm{TP}}{\mathrm{TP}+\mathrm{FP}}$$

(15) $$\mathrm{Recall}=\frac{\mathrm{TP}}{\mathrm{TP}+\mathrm{FN}}$$

where TP is the number of correctly classified positive samples, FP is the number of negative samples classified as positive samples, and FN is the number of positive samples considered harmful. The formula for AP is as follows:

(16) $$\mathrm{AP}=\int_{0}^{1}P\left(R\right)\;dR$$

(17) $$\mathrm{mAP}=\frac{1}{N}\sum_{i=1}^{N}{\mathrm{AP}}_{i}$$

AP50 represents the mean Average Precision calculated at an IoU threshold of 0.5 across all categories. AP (or $AP_{50:95}$) denotes the average of mAP values computed over IoU thresholds ranging from 0.5 to 0.95, with a step size of 0.05. Following the COCO evaluation protocol, use the notations AP and AP50 to refer to these primary summary metrics.

### 4.2. Performance Comparisons

#### 4.2.1. Performance Comparisons on PCB-M

**Figure 8:**
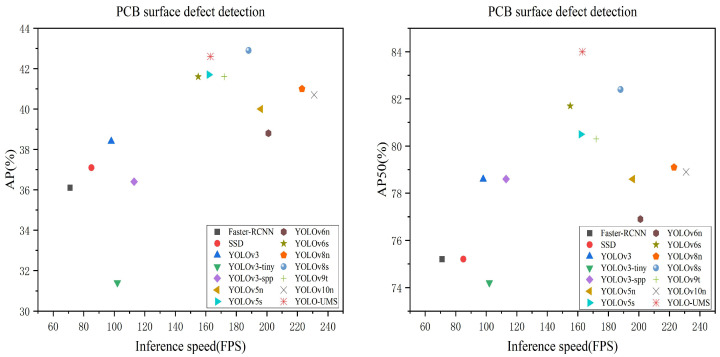
Comparison of different detectors in PCB-M.

The comparison results of the different models on the PCB-M dataset are shown in Table 2 and Figure 8. To ensure the fairness of the comparison, all models were trained from scratch without pre-trained models and tested on RTX 4090 with batch size = 64, and performance metrics, such as AP, AP50, FLOPs, and FPS, were recorded for each model.

A comprehensive evaluation in Table 2 reveals that YOLO-UMS achieves a superior synergy between detection accuracy and architectural efficiency compared to state-of-the-art detectors. Firstly, regarding the accuracy-complexity trade-off, while YOLOv8s maintains a marginal lead in AP (42.9%), its computational demand (23.5 G FLOPs) is 3.17 times higher than that of YOLO-UMS (7.4 G). This demonstrates that our model effectively prunes redundant parameters without sacrificing essential feature representation, making it highly suitable for resource-constrained industrial edge devices.

Secondly, in terms of small object detection ($AP_{S}$), which is critical for identifying hairline cracks and micro-short circuits in PCBs, YOLO-UMS achieves 4.8%, significantly outperforming YOLOv5n (1.8%) and YOLOv3-tiny (1.6%). This improvement suggests that our [specific module name] enhances the spatial resolution of deep features.

Lastly, while YOLOv10n offers a higher inference speed (231 FPS), its precision lags behind at 40.7% AP. YOLO-UMS maintains a robust 163 FPS, which comfortably exceeds the real-time requirements of high-speed PCB assembly lines while providing a more reliable detection rate (42.6% AP), thereby minimizing the risk of pseudo-defects in automated quality control.

#### 4.2.2. Comparative Experiments on the Computational Effort of Feature Extraction Modules

**Figure 9:**
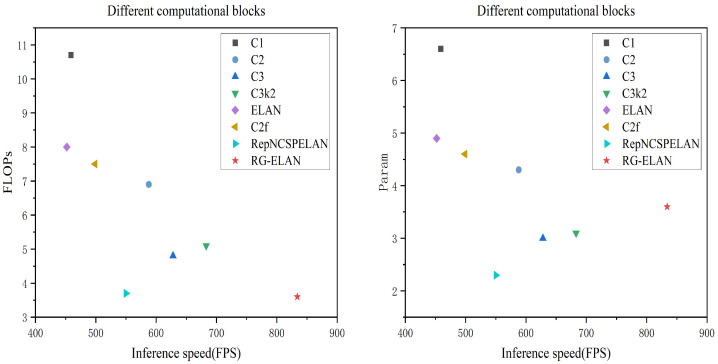
Comparative experiments on the computational effort of feature extraction modules.

Comparison experiments were conducted between the RG-ELAN module and other common YOLO modeling computational blocks (including C1, C2, C3, ELAN, and RepNCSPELAN, etc.) in terms of FPS, computational volumes (FLOPs), and parametric quantities, and the results are shown in Table 3 and Figure 9. These data clearly show that the RG-ELAN module exhibits significant advantages in lightweight design. Specifically, RG-ELAN has the highest frame rate among all the compared modules with 834 FPS, and its computation is only 3.6 GFLOPs, which is not much different from RepNCSPELAN, with the smallest number of references (2.3M). At the same time, it substantially outperforms the complexity of modules such as C1, C2, and ELAN. In addition, RG-ELAN also exhibits a better balance in terms of frame rate and computational efficiency compared to the C2f and C3 modules.

This result demonstrates that RG-ELAN can significantly improve the speed of small target detection in resource-constrained environments and can effectively control the complexity of the model, thus satisfying the dual needs of efficiency and lightweight in practical applications. It further demonstrates the rationality and superiority of the design of the RG-ELAN module when targeting lightweight, small target detection tasks.

### 4.3. Ablation Study

#### 4.3.1. Ablation Study of Network Designed

**Figure 10:**
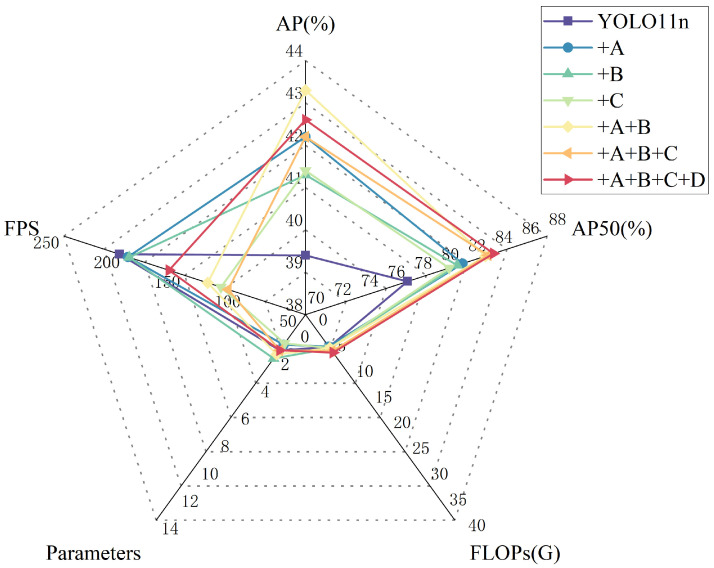
Ablation study results.

In this paper, we perform ablation experiments on the locally-constructed PCB-M dataset to evaluate the network. The experiments incorporate the improved Figure 1 and use the n-version of YOLO11 as the baseline.The effects of the different parts on the model are shown in Table 4 and Figure 10 below. For the designed RG-ELAN module, AIFI was used instead of SPPF in the YOLO11 backbone, and ablation experiments were carried out using the innovative UMSFPN neck and the CPCA in the head. A denotes RG-ELAN, B denotes AIFI, C denotes UMSFPN, and D denotes CPCA.

Using the new RG-ELAN module for the backbone in the PCB-M dataset resulted in a 2.8% improvement in AP and a 4.1% improvement in AP50. Despite the loss in FPS, both FLOPs and the number of parameters were reduced, which is entirely in line with the original intent of the model refinement to improve leakage detection. The high-efficiency layer has a reparameterized aggregation network, enhancing the feature reuse capability. Using the AIFI module instead of the original model SPPF for feature refinement improves AP by 1.9% and AP50 by 3.7% with only a 0.4 G increase in computation. Suppose the neck is replaced with a model in the UMSFPN framework. In that case, it improves 2% AP and 3.1% AP50 compared to the baseline, which proves that the performance of UMSFPN in multi-scale feature fusion is favorable for improving the model. If RG-ELAN and AIFI are used in the trunk and UMSFPN is used in the neck, the improved model achieves 7.1 GFLOPs and 163 FPS with 42.2% AP and 83.6% AP50. Suppose the CPCA is used in the head on top of this. In that case, an AP of 42.6% and an AP50 of 84% can be achieved, which is a 3.2% improvement in AP and a 6.4% improvement in AP50 compared to the baseline, achieving a better balance between speed and accuracy.

#### 4.3.2. UMSFPN Analysis

In this section, an attempt is made to perform ablation experiments on UMSFPN and its improved modules. The neck structure is replaced with different algorithmic models to demonstrate UMSFPN’s plug-and-play capability. The ablation experiments are performed on different datasets to check UMSFPN’s generalization performance.

This paper conducts ablation experiments with other feature fusion components, as shown in Table 5, to verify the effectiveness of weighted feature fusion using BiFPN at the neck.

First, weight is employed as the fusion strategy to achieve 43.1% AP and 83.5% AP50 with 7.5 GFLOPs and 126 FPS. Subsequently, the adaptive fusion strategy is introduced instead of weight. Although its FLOPs are on par with weight (7.5G), the FPS decreases to 100, and the inference latency increases significantly. Meanwhile, the AP and AP50 of the adaptive method decreased to 42.9% and 82.7%, respectively, indicating that this strategy is not superior to weight in terms of accuracy and efficiency. Furthermore, concat was tried as a new fusion strategy. Concat achieved 43.1% AP and 83.1% AP50 with slightly lower 7.3 GFLOPs and 111 FPS. Compared to adaptive, concat achieved 43.1% AP and 83.1% AP50 with slightly lower 7.3 GFLOPs and 111 FPS. Compared to adaptive, concat significantly improves the inference speed, but its FPS is somewhat lower, and AP is flat compared to weight. Finally, the BiFPN fusion strategy is introduced. With FLOPs further decreasing to 7.1, BiFPN achieves the highest AP50 (83.6%), which is more conducive to maximizing the performance of the UMSFPN neck of the paper.

**Figure 11:**
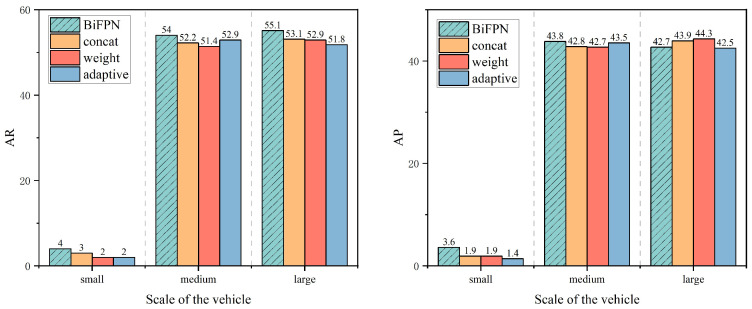
The impact of different fusion strategies on multi-scale object detection performance.

Further analyzing the effect of different fusion strategies on the multi-scale target detection performance, the results of comparing the average recall (AR) and average precision (AP) of BiFPN, concat, weight, and adaptive methods on different scale targets are shown in Figure 11. BiFPN leads the AR in small and medium scale targets with 54% and improves the big scale targets in both AP and AR. The BiFPN method is the most comprehensive fusion strategy in performance performance due to its efficient feature fusion capability and adaptability to multi-scale targets.

Ablation experiments on CSPMS, a new feature extraction module designed in UMSFPN, have been conducted to check its effectiveness. Experiments on the CSPMS module using other feature extraction modules for YOLO models have shown that CSPMS has a higher parameter utilization and accuracy performance than other modules. Table 6 demonstrates the performance of CSPMS compared to other modules.

Table 7 below illustrates the outcomes of using UMSFPN as a plug-and-play module for various models. Initially, UMSFPN was used instead of the popular target detector YOLOv5n neck, and the unified model’s channels were 256. Compared to the previous model, YOLOv5n with UMSFPN had fewer parameters and improved by 1.2% and 0.4% in AP and AP50, respectively, demonstrating UMSFPN’s outstanding performance. Furthermore, the YOLOv9t model also confirms the efficacy of UMSFPN.

Finally, the impact of UMSFPN on the two public datasets, PCB-D and PCB-B, is shown in Table 8 below. UMSFPN improves AP by 0.9% and AP50 by 1.5% on the dataset PCB-D, which suggests that UMSFPN has a good improvement against PCB-D. Similarly, although the progress is not very significant on the PCB-B dataset, it still improves the AP by 0.5% and AP50 by 0.9%. The results of the experiments show that the UMSFPN is robust on different datasets and can improve the detector’s detection accuracy.

#### 4.3.3. Evaluation of the Effectiveness of Attention

**Figure 12:**
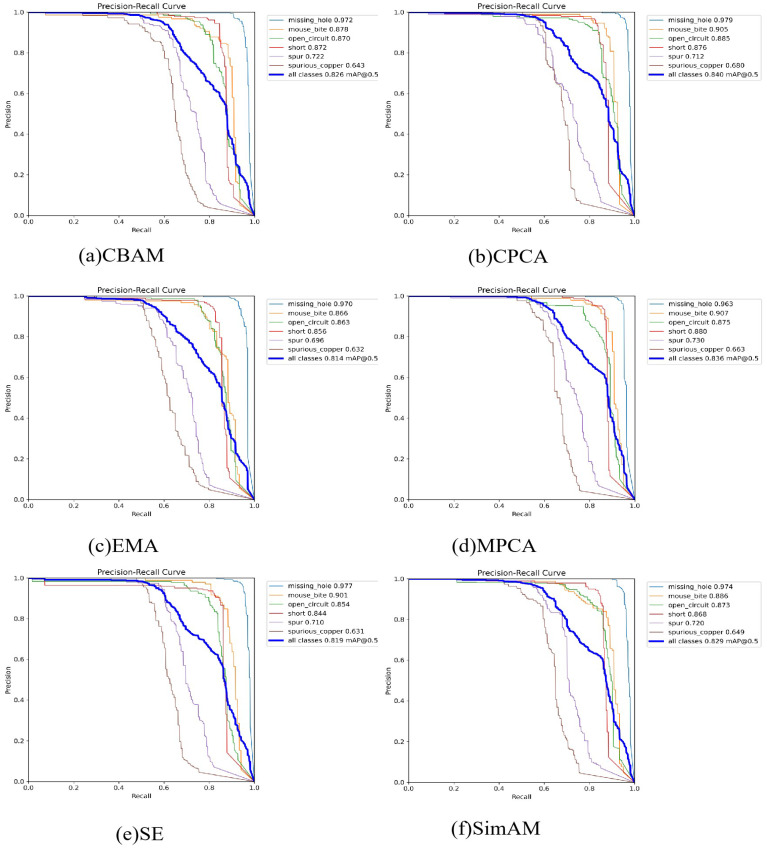
P-R curve of different attention modules.

In this section, ablation experiments with commonly used attentional mechanisms (including SE, EMA, CMBA, MCPA, and SimAM) are conducted to verify the effectiveness of CPCA applied to this improved model. The model without the attention module was used as the baseline model, and different attention modules were added in the last layer for experimental validation, respectively, indicated by red blocks in Figure 1. The experimental results are shown in Table 9, which show that CPCA’s AP performance is better than other attention mechanisms. At the same time, the computational consumption is almost negligible, and the AP50 is improved by 0.4% compared to that of the baseline model. It is also interesting that CPCA improves recall (R) and excels in detection precision (P). The P-R curves of different attention modules are shown in Figure 12.

Explicitly analyzing the detection performance of different defect types, it can be found that spurious copper and spur are the more difficult types to detect because they have higher uncertainty. On the baseline model, the detection precision performances of spurious copper and spur are 65.6% and 73.7%, respectively, significantly lower than those of other defect types. After introducing the attention mechanism, the detection accuracy of spurious cooper is significantly improved, but the detection performance of spur does not show any significant improvement. The AP50 of CPCA for open-circuit and spurious copper is enhanced by 1.8% and 2.4%, respectively. The detection performance of CPCA for open circuits is improved by 1.2% compared to SimAM. The detection performance of CPCA for open circuits is 1.2% better than that of SimAM, and the detection performance of CPCA for spurious copper is 1.7% better than that of MPCA. CPCA performs excellently in most defect types and is more suitable for this model.

The improved model with and without CPCA is applied to the two publicly available datasets. The effects of CPCA on PCB-D and PCB-B are shown in Table 10. CPCA improves 2% AP and 2.2% AP50 on PCB-B, which indicates that CPCA has a good improvement in the PCB-B dataset. In contrast to PCB-M, PCB-D has a lot of data and a high level of data identifiability. The degree is high. Although the progress is insignificant, CPCA still improves by 0.2%m AP and 0.5%AP50. The experimental results show that CPCA is relatively robust in different datasets, especially in defect images obtained by linear scanning CCDs, indicating that this attention mechanism enhances the detection performance of this model.

### 4.4. Visualizations

**Figure 13:**
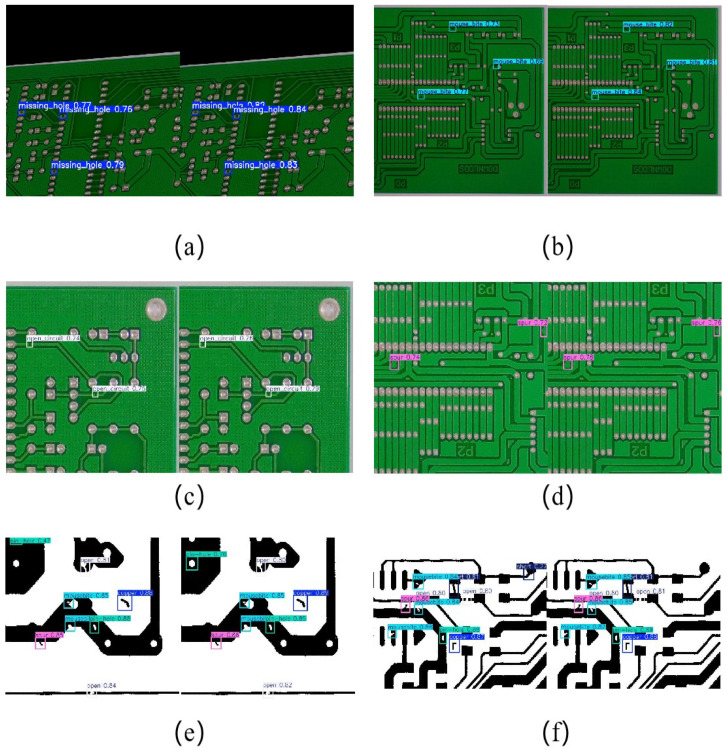
Results of tests on different datasets.

The detection performance of YOLO-UMS in several datasets is shown in Figure 13. The figures compare the detection structure of the baseline: (a) and (b) are the detection results at PCB-M, (c) and (d) are the detection results at PCB-B, and (e) and (f) are the detection results at PCB-D. The left side of each plot shows the detection results of YOLO11, and the right side shows the detection results of YOLO-UMS. For small target detection like defects, YOLO-UMS detects them more accurately than the original model. From Figure 13f, it is evident that the enhanced YOLO-UMS model eliminates the false detections present in the original model when it comes to identifying dense, tiny targets.

**Figure 14:**
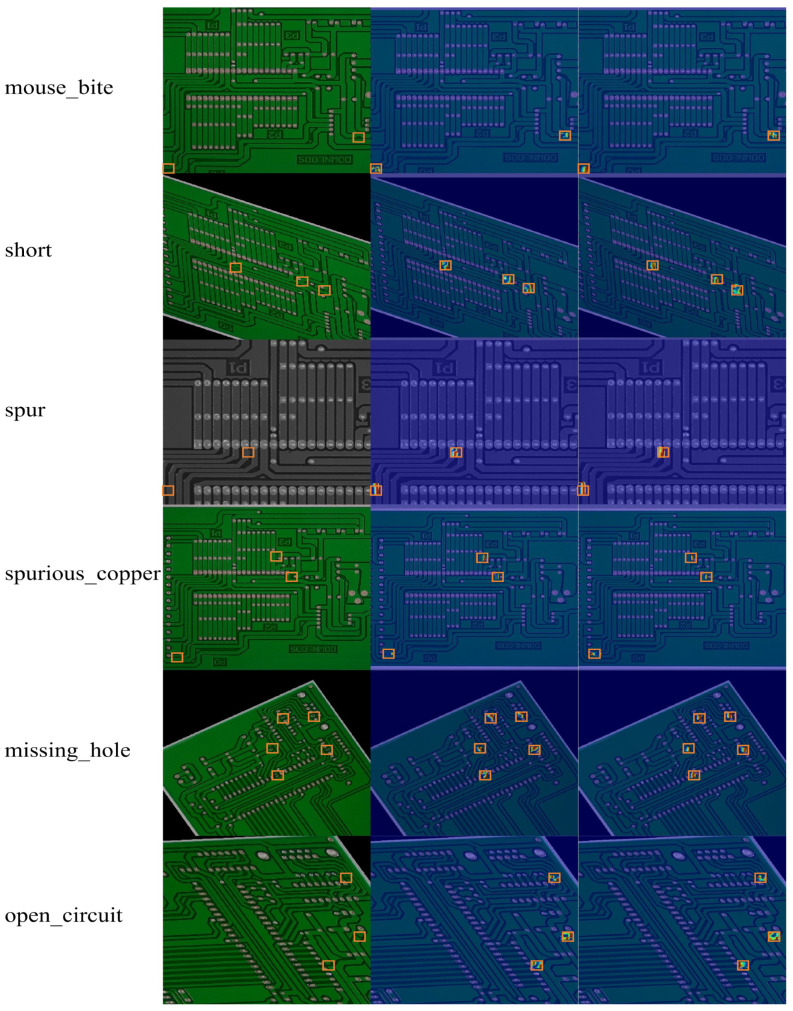
Visualization of different defect categories.

To verify the feasibility of the proposed UMSFPN neck, this paper carries out a randomized test experiment to randomly select one image from each type of defective image in the test set and simulate detection using the proposed YOLO-UMS network model with or without UMSFPN. An efficient neck structure requires high prediction accuracy and the effectiveness of the extracted features. In this paper, we visualize the feature extraction capability of PCB surfaces for six types of defects, and the detection results and visualization effects are shown in Figure 14. The first in each group is the original image, the second is the YOLO-UMS without UMSFPN, and the third is the YOLO-UMS with UMSFPN. From the second and third columns in the figure, we can compare that for the network without UMSFPN, the detection area for defects is lighter in color. With the introduction of UMSFPN, the network can focus more on the distinctive features of the defects to learn where the features are concentrated, reducing the uncertainty of the boundaries and exploiting the central features of the target.

**Figure 15:**
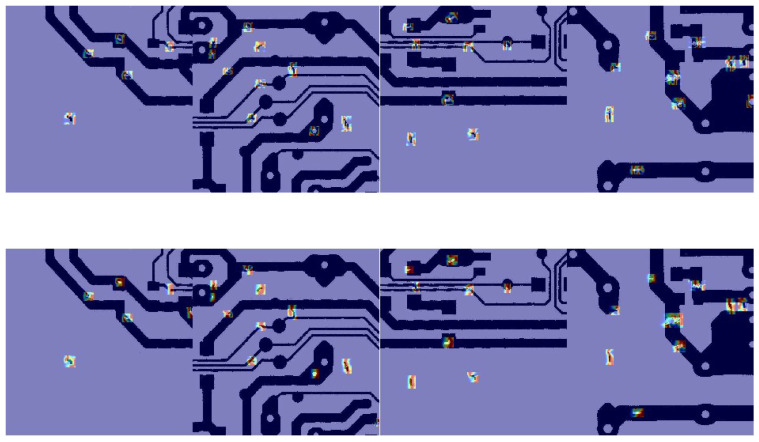
Visualization on dataset PCB-D.

Additionally, the improvement of UMSFPN on PCB-D is demonstrated in Figure 15. The first row shows the visualization of YOLO-UMS without UMSFPN, while the second row illustrates the enhancement brought by UMSFPN. In scenarios with densely distributed defective targets, the proposed method is more capable of focusing on the valuable target information, highlighting the robustness of the UMSFPN against poor feature maps. As shown in the figure, when dealing with dense targets, UMSFPN achieves higher detection accuracy and more accurately extracts target features, as evidenced by the darker regions at the center of the targets.

## 5. Conclusions

In this paper, a UMSFPN is proposed, which not only improves the accuracy of small target detection but maintains a low computational overhead, which is suitable for complex practical application environments. Meanwhile, the RG-ELAN feature extraction module is designed to reduce the computational overhead using the reparameterization technique, significantly enhancing the detection of small targets. The introduced AIFI module improves the feature interaction capability and fine-grained feature expression of the model, and the CPCA attention mechanism further enhances the model’s sensitivity to features of targets of different sizes, significantly improving the small target detection accuracy. Based on this, the YOLO-UMS PCB surface defect detector is constructed, and without relying on additional data pre-training, YOLO-UMS achieves 42.6% AP and 84.0% AP50 with the same amount of computation, which successfully achieves a better balance between speed and accuracy, and it is more suitable for PCB surface defect detection than other detectors.

The experimental results show that YOLO-UMS exhibits significant performance improvement on the self-collected PCB-M dataset, with YOLO-UMS improving by 3.2% and 6.4% in AP and AP50, respectively, compared to the baseline YOLO11. In addition, UMSFPN performs outstandingly across different models and different datasets and can help YOLO v5 and YOLO v9t to improve 1.2% AP and 0.7% AP, respectively, and 0.5% AP and 0.9% AP on the datasets PCB-B and PCB-D, respectively. These results validate the superiority of YOLO-UMS in detecting surface defects on PCBs and broad applicability. At the same time, YOLO-UMS has good generalization ability and can provide efficient and accurate inspection results in various complex scenarios.

Although YOLO-UMS has achieved good results on multiple datasets, its robustness under extreme environments (e.g., severe occlusion and intense illumination changes) still needs further verification. Future research could focus on further optimizing the inference speed in higher resolution and complex scenes, especially for applications on low-power devices. In addition, dynamic model tuning strategies based on environment adaptive mechanisms will be explored to ensure the best performance of YOLO-UMS in various application scenarios. Finally, YOLO-UMS can be further optimized to support better real-time detection and adapt to different hardware platforms (e.g., embedded devices and mobile devices).

## Figures and Tables

**Table 1:** Initialization parameters of our method.

Parameters	Value
Batch size	64
Image size	640 × 640
Optimizer	SGD
Weight decay	0.0005
Learning rate	0.01
Momentum	0.937
Epochs	300

**Table 2:** Comparison of different detectors in PCB-M.

Method	Input Size	AP(%)	AP50(%)	${\mathrm{AP}}_{S}$(%)	${\mathrm{AP}}_{M}$(%)	${\mathrm{AP}}_{L}$(%)	FLOPs	FPS
Faster-RCNN	640 × 640	36.1	75.2	2.5	40.2	35.4	60.2 G	71
SSD	640 × 640	37.1	75.9	3.2	41.1	35.9	41.2 G	85
YOLOv3	640 × 640	38.4	78.6	4.8	39.6	36.3	154.6 G	98
YOLOv3-tiny	640 × 640	31.4	74.2	1.6	32.5	30.0	12.9 G	102
YOLOv3-spp	640 × 640	36.4	78.6	2.5	38.2	34.2	155.5 G	113
YOLOv5n	640 × 640	40.0	78.6	1.8	41.0	38.5	6 G	196
YOLOv5s	640 × 640	41.7	80.5	3.1	42.1	41.9	18.9 G	162
YOLOv6n	640 × 640	38.8	76.9	3.1	39.6	37.8	11.7 G	201
YOLOv6s	640 × 640	41.6	81.7	4.8	41.3	44.4	42.9 G	155
YOLOv8n	640 × 640	41.0	79.1	1.9	41.8	40.0	8.7 G	223
YOLOv8s	640 × 640	42.9	82.4	3.3	43.8	41.9	23.5 G	188
YOLOv9t	640 × 640	41.6	80.3	1.0	42.4	41.2	6.6 G	172
YOLOv10n	640 × 640	40.7	78.9	3.1	41.5	39.8	6.7 G	231
YOLO-UMS	640 × 640	42.6	84.0	4.8	42.4	42.8	7.4 G	163

**Table 3:** Comparative experiments on the computational effort of feature extraction modules.

Name	All_Time (s)	Mean_Time (s)	FPS	FLOPs	Params
C1	4.3509	0.00128	459	10.7 G	6.6 M
C2	3.39778	0.0017	588	6.9 G	4.3 M
C3	3.18075	0.00159	628	4.8 G	3.0 M
C3k2	2.92906	0.00146	683	5.1 G	3.1 M
ELAN	4.42797	0.00221	452	8.0 G	4.9 M
C2f	4.00482	0.00200	499	7.5 G	4.6 M
RepNCSPELAN	3.63498	0.00182	550	3.7 G	2.3 M
RG-ELAN	2.39777	0.00120	834	3.6 G	2.2 M

**Table 4:** Ablation study results.

Methods	AP (%)	AP50 (%)	FLOPs	Params	FPS
YOLO11n	39.4	77.6	6.3 G	2.6 M	204
+A	42.2 (+2.8)	81.7 (+4.1)	6.2 G	2.2 M	197
+B	41.3 (+1.9)	81.3 (+3.7)	6.6 G	3.2 M	196
+C	41.4 (+2.0)	80.7 (+3.1)	6.5 G	2.1 M	120
+A+B	43.3 (+3.9)	83.4 (+5.8)	6.5 G	2.9 M	131
+A+C	42.8 (+3.4)	82.9 (+5.3)	6.8 G	2.0 M	172
+A+B+C	42.2 (+2.8)	83.6 (+6.0)	7.1 G	2.6 M	114
+A+B+C+D	42.6 (+3.2)	84.0 (+6.4)	7.4 G	2.6 M	163

**Table 5:** Results of ablation experiments with other characteristic fusion components.

Fusion Method	Weight	Adaptive	Concat	BiFPN
FOLPs	7.5G	7.5G	7.3G	7.4G
AP (%)	43.1	42.9	43.1	42.6
AP50 (%)	83.5	82.7	83.1	84.0
FPS	126	100	111	163

**Table 6:** The impact of different computational blocks on the UMSFPN.

Blocks	Param	FLOPs	AP (%)	AP50 (%)
C2f	2.7 M	7.8 G	41.7	82.5
C3	2.5 M	7.2 G	42.1	83.0
C3k2	2.6 M	7.4 G	42.2	82.9
CSPMS	2.6 M	7.4 G	42.6	84.0

**Table 7:** Plug-and-play UMSFPN neck performance on different detectors.

Method	UMSFPN	Param	AP (%)	AP50 (%)
YOLOv5n	✕	2.1 M	40.2	79.2
	✓	1.7 M	41.4	79.6
YOLOv9t	✕	1.7 M	41.5	80.9
	✓	8.5 M	42.2	82.4

**Table 8:** The plug-and-play UMSFPN neck performed differently on different datasets.

Method	UMSFPN	AP (PCB-M) (%)	AP50 (PCB-M) (%)	AP (PCB-B) (%)	AP50 (PCB-B) (%)	AP (PCB-D) (%)	AP50 (PCB-D) (%)
YOLO-UMS	✕	42.5	83.5	68.2	98.3	78.6	97.1
	✓	42.6	84.0	68.7	99.2	79.5	98.6
Variation		+0.1	+0.5	+0.5	+0.9	+0.9	+1.5

**Table 9:** Improvements to different attention modules.

Method	AP50 (%)	P (%)	R (%)	Missing Hole (%)	Mouse Bite (%)	Open Circuit (%)	Short (%)	Spur (%)	Spurious Cooper (%)
Baseline	83.6	93.1	77.0	97.6	90.1	86.7	88.1	73.7	65.6
CBAM	82.6	89.6	76.7	97.2	87.8	87.0	87.2	72.2	64.3
EMA	81.4	94.3	72.8	97.0	86.6	86.3	85.6	69.6	63.2
SE	81.9	91.0	76.2	97.7	90.1	85.4	84.4	71.0	63.1
SimAM	82.9	89.8	87.9	97.4	88.6	87.3	86.8	72.0	64.9
MPCA	83.6	92.2	77.2	96.3	90.7	87.5	88.0	73.0	66.3
CPCA	84.0	94.6	77.4	97.9	90.5	88.5	87.6	71.2	68.0

**Table 10:** Improvements of the CPCA on different datasets.

Method	CPCA	AP (PCB-M) (%)	AP50 (PCB-M) (%)	AP (PCB-B) (%)	AP50 (PCB-B) (%)	AP(PCB-D) (%)	AP50 (PCB-D) (%)
YOLO-UMS	✕	42.2	83.6	66.7	97.0	79.3	98.1
	✓	42.6	84.0	68.7	99.2	79.5	98.6
Variation		+0.4	+0.4	+2.0	+2.2	+0.2	+0.5

## Data Availability

The data presented in this study are available on request from the corresponding author.
